# Identification of immune‐associated signatures and potential therapeutic targets for pulmonary arterial hypertension

**DOI:** 10.1111/jcmm.17962

**Published:** 2023-09-27

**Authors:** Xu He, Jiansong Fang, Mingli Gong, Juqi Zhang, Ran Xie, Dai Zhao, Yanlun Gu, Lingyue Ma, Xiaocong Pang, Yimin Cui

**Affiliations:** ^1^ Department of Pharmacy Peking University First Hospital Beijing China; ^2^ Institute of Clinical Pharmacology Peking University First Hospital Beijing China; ^3^ Science and Technology Innovation Center Guangzhou University of Chinese Medicine Guangzhou China; ^4^ School of Pharmacy Xu Zhou Medical University Xuzhou China

**Keywords:** bioinformatic analysis, immune, pulmonary arterial hypertension, therapeutic drugs

## Abstract

Pulmonary arterial hypertension (PAH) comprises a heterogeneous group of diseases with diverse aetiologies. It is characterized by increased pulmonary arterial pressure and right ventricular (RV) failure without specific drugs for treatment. Emerging evidence suggests that inflammation and autoimmune disorders are common features across all PAH phenotypes. This provides a novel idea to explore the characteristics of immunological disorders in PAH and identify immune‐related genes or biomarkers for specific anti‐remodelling regimens. In this study, we integrated three gene expression profiles and performed Gene Ontology (GO) and KEGG pathway analysis. CIBERSORT was utilized to estimate the abundance of tissue‐infiltrating immune cells in PAH. The PPI network and machine learning were constructed to identify immune‐related hub genes and then evaluate the relationship between hub genes and differential immune cells using ImmucellAI. Additionally, we implemented molecular docking to screen potential small‐molecule compounds based on the obtained genes. Our findings demonstrated the density and distribution of infiltrating CD4 T cells in PAH and identified four immune‐related genes (*ROCK2*, *ATHL1*, *HSP90AA1* and *ACTR2*) as potential targets. We also listed 20 promising molecules, including TDI01953, pemetrexed acid and radotinib, for PAH treatment. These results provide a promising avenue for further research into immunological disorders in PAH and potential novel therapeutic targets.

## 1 INTRODUCTION

Pulmonary arterial hypertension (PAH) is a progressive cardiopulmonary disorder with complex and multifactorial phenotypes that can lead to increased pulmonary vascular resistance and death. Vascular pathology of PAH consists of arterial, medial and intimal remodelling, plexiform lesions and fragmentation of the elastic lamina. Effective treatments ideally target remodelling of pulmonary arteries as well as vasoconstriction.^1^ Vasoconstriction is an important component of PAH, but its role as an inciting event or a complication of pulmonary vascular remodelling is still uncertain. Current approved drugs for PAH, including prostacycline analogues and prostacycline receptor agonists, endothelin receptor antagonists, phosphodiesterase inhibitors and guanylate cyclase agonists, focus on dilatation of the pulmonary arterial vasculature and reducing pulmonary vascular resistance, which can only ameliorate symptoms but cannot completely reverse pulmonary vascular remodelling and improve overall survival.^2^, ^3^ Although current treatments for PAH can improve survival, they are not considered disease‐modifying as the mean 5‐year survival rate of PAH patients still remains at only 57%–59%.^4^ The reason for this is that pathological mechanisms of inflammation and immune dysregulation have not been adequately addressed.^5^ As a result, there is an urgent need for more insights into the pathogenesis of PAH, immune dysregulation and the development of specific anti‐remodelling regimens to explore new therapies.

Although different forms of PAH could reflect distinct physiopathological mechanisms, current evidence strongly suggests that dysimmunity is a common feature of all forms.^6^, ^7^, ^8^ In particular, it is now widely accepted that immunological disorders contribute to both disease susceptibility and the progression of vascular remodelling in PAH.^6^ There is a positive correlation between the degree of pulmonary perivascular inflammation, vascular intimal/medial/adventitial thickness and mean pulmonary arterial pressure. Various inflammatory and immune markers are correlated with the hemodynamics or prognosis of PAH patients.^9^, ^10^ These findings highlight that inflammation could be involved in pulmonary vascular remodelling and PAH development.^11^ Increasing evidence of the pathological role of immune cells in innate and adaptive immunity leads to many promising preclinical studies. IL‐6‐specific antagonist treatment reversed sugen/hypoxia‐induced PAH and MCT‐PAH in rat models.^12^ T‐cell lymphopenia in association with vascular endothelial growth factor receptor 2 (VEGFR2) blockade resulted in periarteriolar inflammation with macrophages and B cells even prior to vascular remodelling and elevated pulmonary pressures. And immune reconstitution prevented early inflammation and attenuated PAH development.^13^ Activation of endogenous retrovirus sequences and their gene products could contribute to the chronic inflammatory and altered immune state related to the vascular remodelling in PAH. This activation may occur through activating monocytes, which leads to elevated production of cytokines such as TNFα, IL6 and IL1β, as well as SAMHD1. Additionally, this activation may also activate B cells, which could be responsible for the production of SAMHD1 antibodies in tertiary lymphoid tissue in the lungs.^5^ These preclinical studies, in turn, are contributing to innovative clinical trials. The TRANSFORM‐UK trial (NCT 02676947) has been completed, in which PAH patients are being treated with anti‐IL‐6, and 21 patients received the drug with good safety.^14^ A randomized multicentre placebo‐controlled clinical trial testing B‐cell depletion with rituximab to treat systemic sclerosis‐associated PAH^15^ has also been conducted. To lead the way for specific targeted therapeutic approaches intervening in the affected homeostasis of innate and adaptive immunity, a deeper insight into the immune‐related molecular mechanisms and interactions of the depicted contributors to the pathophysiology of PAH is essential. Gene sequencing, high‐throughput technologies and bioinformatics methods are widely applied to screen new biomarkers or potential drug targets. For example, two new candidate genes—FBLN2 and PDGFD—with known functions in vasculogenesis and remodelling were identified by variant analysis by a large international consortium, and trio analysis predicted that 15% of paediatric IPAH may be explained by de novo variants.^16^

Here, we investigated the signatures of tissue‐infiltrating immune cells during the development of proliferative remodelling in PAH by integrating transcriptomic data of PAH lung tissues, followed by the validation of immune cell abundance and those genes in an experimental PAH model, which would enrich our understanding of the roles of inflammation and immune mechanisms in PAH progression and help to discover new potential biomarkers and treatment targets.

## 2 MATERIALS AND METHODS

### 2.1 Data collecting and processing

Gene expression profiles of human lung tissue with or without PAH were acquired from the GEO database (https://www.ncbi.nlm.nih.gov/geo/). GSE53408, GSE117261 and GSE15197, containing 96 samples of PAH and 49 normal controls, were included in this study. To reduce the bias and variability inherent in these three different sequencing results, we integrated and processed the three data sets using the sva package in the R language, which supports using the sva function of proxy variable estimation, the ComBat function of adjusting known batch effects directly and the Fsva function of adjusting batch and latent variables in predictive problems. The limma Bioconductor package (R version 3.5.1) was used to make class comparison analysis for DEGs between PAH samples and normal samples, and DEGs with a threshold of *p* < 0.05 and |log2FC| >1 were selected for subsequent analysis.

### 2.2 GO and KEGG pathway enrichment

The Database for Annotation, Visualization and Integrated Discovery (DAVID, https://david.ncifcrf.gov/) was used for GO biological pathway and cellular component enrichment.^17^ The GO plot package was used to combine and integrate the expression data with the results of the DAVID analysis. KEGG pathway annotation and analysis of DEGs, which can determine the major metabolic and signal transduction pathways involved in these genes, were analysed by Cluster profiler in R software, performing statistical analysis and visualization of functional clustering of gene collections.^18^ *p* values <0.05 were considered statistically significant.

### 2.3 Evaluation of tissue‐infiltrating immune cells

CIBERSORT is a bioinformatics tool that uses the deconvolution method to characterize cell subsets of interest in high‐dimensional genomic data derived from bulk tissue samples, which provides an estimate of the abundance of member cell types in a mixed cell population using gene expression data. It is used to estimate the proportion of 22 types of tissue‐infiltrating immune cells (TIICs) in each sample, such as naive B cells, plasma cells, CD4+ resting memory T cells, Tregs, activated natural killer cells, monocytes, M0 macrophages, resting dendritic cells, activated mast cells, eosinophils and neutrophils.^19^, ^20^ Processed GEPs were transformed into an abundance of 22 TIICs. The relative expression of 22 TIICs in each sample was determined. Significant results with a threshold of *p* < 0.05 were selected for further analysis.

### 2.4 Construction of the PPI network

In the present study, the PPI network of DEGs was constructed by using the Retrieval of Interacting Genes (STRING) database (http://string‐db.org/). Analysing the functional interactions between genes may provide insights into the mechanisms of PAH development. Herein, the PPI network of DEGs was built with a threshold interaction score of 0.4, which was visualized by the Cytoscape software (version 3.7.2). Cytoscape software is a very formidable tool for visualizing the interrelationships among a set of genes or proteins. The hub genes were screened by PPI and the CytoHubba plugin.

### 2.5 Classification by machine learning

Pearson correlation analysis was used to wipe off the high‐autocorrelation and low correlation between the phenotypes of normal tissue and PAH tissue and gene signatures. The phenotypes of normal tissue and PAH tissue were set as ‘0’ and ‘1’, respectively. The correlation coefficient <0.1 was eliminated, and if the autocorrelation was more than 0.9, the gene signature with a lower correlation was removed. Then the stepwise regression approach was further used to filter the remaining gene signatures, which considered variable size, significance and contribution. Every new regression equation was employed with a significance test to assess the addition of each new gene signature. When there were no novel signatures inputted or removed, the process was terminated. After filtration, the selected descriptors served as signatures to establish the prediction models. The normal and PAH tissues were divided into training and test groups according to a ratio of 3:1. With the remaining gene signatures, logistic regression (LR), naive Bayes (NB) and support vector machine (SVM) algorithms were used to establish PAH and normal tissue classification models by Orange Canvas 3.13 (26). The performance of the three models was assessed by fivefold random sampling and test set validation by calculating the parameters of AUC, precision, specificity and sensitivity.

### 2.6 Estimation correlation between infiltrating immune cells and hub genes

The normalized gene matrix was analysed by ImmuCellAI online (http://bioinfo.life.hust.edu.cn/ImmuCellAI), which is a gene set signature‐based method for estimating the abundance of tissue‐infiltrating immune cell populations from transcriptomic data.^21^ Compared with CIBERSORT mentioned above, the abundance of 18T‐cell subsets could be evaluated by ImmuCellAI, such as Th17, Th1 and Treg. The immune cell abundance in all samples was estimated, and Spearman was used to evaluate the correlation between the obtained immune cell content and hub genes in each sample. The results were visualized by the ggplot2 package within R.

### 2.7 Animal models of pulmonary arterial hypertension and hemodynamic measurements

Twenty‐four male Sprague–Dawley rats (220–250 g) were randomly assigned to the following two groups (*n* = 12 per group): The PAH group was induced with a single intraperitoneal injection of MCT (60 mg/kg) on Day 0; the parallel group of control rats was injected with an equal volume of saline (Nor). On Day 28, we anaesthetized the rats and measured their pulmonary artery pressure (PAP) through right‐sided heart catheterization. After euthanizing the rats, we weighed the wall of the right ventricular (RV) and left ventricle plus the ventricular septum (LV + S), calculated the ratio of RV/LV + S to evaluate the remodelling of the right ventricle, and collected lung tissues for haematoxylin and eosin (HE) staining, immunohistochemistry (IHC) staining and immunofluorescence staining.

Ten male C57BL/6J mice were randomly divided into two groups (*n* = 5 per group): normoxia group and hypoxia group. These mice were exposed to normoxia (20% O_2_) or hypoxia (10% O_2_) for 14 days. The body weight of the mice was recorded every 3 days. At the end, the mice were anaesthetized (200 mg/kg tribromoethanol) to measure the PAP by using the Millar catheter‐transducer (Millar Instruments). Then the mice were euthanized, and the lung tissues were collected for q‐PCR, Masson's trichrome staining and IHC staining.

### 2.8 Haematoxylin and eosin staining, immunohistochemistry, Masson's trichrome staining and multicolor immunofluorescence staining

We followed the methods of Xiang‐Guang Wu et al.^22^ The left lungs of rat models were embedded in paraffin, sectioned at 5‐μm thickness sections and then stained with haematoxylin and eosin. For immunohistochemistry staining, briefly, the paraffin‐embedded samples were sliced into 8 μm sections. Antigen retrieval was performed using the target retrieval solution Tris‐EDTA buffer in a microwave oven for 10–15 min. The sections were placed in 3% H_2_O_2_ for 30 min to block the endogenous peroxidase activity. 3% BSA was added to evenly cover the tissues to block non‐specific binding for 30 min. The tissues were incubated with antibodies against ROCK2 (1:200, 21,645‐1‐AP, Proteintech) overnight at 4°C, and immune detection was performed using DAB kits (G1212‐200T, Servicebio). For Masson's trichrome staining, the paraffin sections were dewaxed and hydrated using a series of solutions, followed by staining with Masson A (overnight), B (1 min), C (1 min), D (6 min), E (1 min) and F (30 s) solutions (G1006, Servicebio). The slices were then rinsed with 1% glacial acetic acid and dehydrated with anhydrous ethanol before being cleared and sealed with neutral gum. Finally, microscope inspection, image acquisition and analysis were performed.

For multicolor immunofluorescence staining, briefly, the paraffin‐embedded samples were sectioned at 4 mm thickness. Tris‐EDTA buffer (pH 8.0) was used to recover antigen with a pressure cooker for 10–15 min. 3% BSA was added to evenly cover the tissues to block non‐specific binding for 30 min. Subsequently, the sections were incubated with the anti‐CD4 Rabbit pAb (1:3000, GB13064‐2, Servicebio) in a humidified chamber overnight at 4°C, followed by washing and covering the objective tissue with secondary antibody in dark conditions for 50 min at room temperature. Then the similar experimental methods were used for subsequent staining operations. Anti‐CD3 Rabbit mAb (1:1000, GB13014, Servicebio) and ROCK2 (middle) polyclonal antibody (1:200, 21,645‐1‐AP, Proteintech) were used as the first antibodies. The nucleus is blue by labelling with DAPI; positive cells are green, red, pink or purplish red according to the fluorescent labels used; and images were obtained by fluorescence microscopy (Leica DM6).

### 2.9 Quantitative real‐time polymerase chain reaction

Total RNA from the lungs of the PAH model and controls was isolated using TRIzol, and the extracted RNA was reverse transcribed into complementary DNA (cDNA) using the RT First Strand cDNA Synthesis Kit (Servicebio). Quantitative real‐time polymerase chain reaction (q‐PCR) was carried out using SYBR Green qPCR Master Mix (Servicebio) to detect the expression of the genes. GAPDH was employed as a reference control. The primer sequences and the thermal protocol were provided in File S1.

### 2.10 Molecular docking

Molecular docking is a widely used method of exploring the binding modes of small molecules within protein‐ligand complexes for structure‐based virtual screening. Two docking algorithms, LibDock and CDOCKER of DISCOVERY STUDIO 2018 performed in molecular docking analysis. LibDock, a site‐feature docking algorithm developed by Diller and Merz, can easily provide a rapid virtual screening campaign on millions of compounds by matching the physicochemical properties of the compounds to the polar and polar features in the protein binding sites.^23^ CDOCKER is another powerful docking method that is CHARMm‐based and has been validated for generating highly accurate docked poses.^24^ The crystal structure of ROCK2 was acquired from the Protein Data Bank (PDB ID: 6ED6). The interaction energy for every final pose of ligands with CHARMm energy was computed, and the top scoring poses are retained. The structure of ROCK2 was prepared by removing water and adding hydrogen to a clean protein module. The compounds were also prepared by adding hydrogen conversing into 3D structures and pH‐based ionization. We used molecular docking DS 2016 to identify potential ROCK2 inhibitors by screening the Clinical & FDA‐approved Drug Library (https://www.apexbio.cn/discoveryprobetm‐clinical‐fda‐approved‐drug‐library.html). The crystal structure of ROCK2, complexed with *N*‐[(2,3‐dihydro‐1,4‐benzodioxin‐5‐yl) methyl]‐4‐(pyridin‐4‐yl) benzamide, was retrieved from the Protein Data Bank (PDB ID: 6ED6). Protein preparation was conducted by removing the water and co‐crystallized N‐[(2,3‐dihydro‐1,4‐benzodioxin‐5‐yl) methyl]‐4‐(pyridin‐4‐yl) benzamide, adding hydrogens and missing loop regions, calculating protein ionization and protonating the protein structure. The binding site was defined by the primary ligand.

**FIGURE 1:**
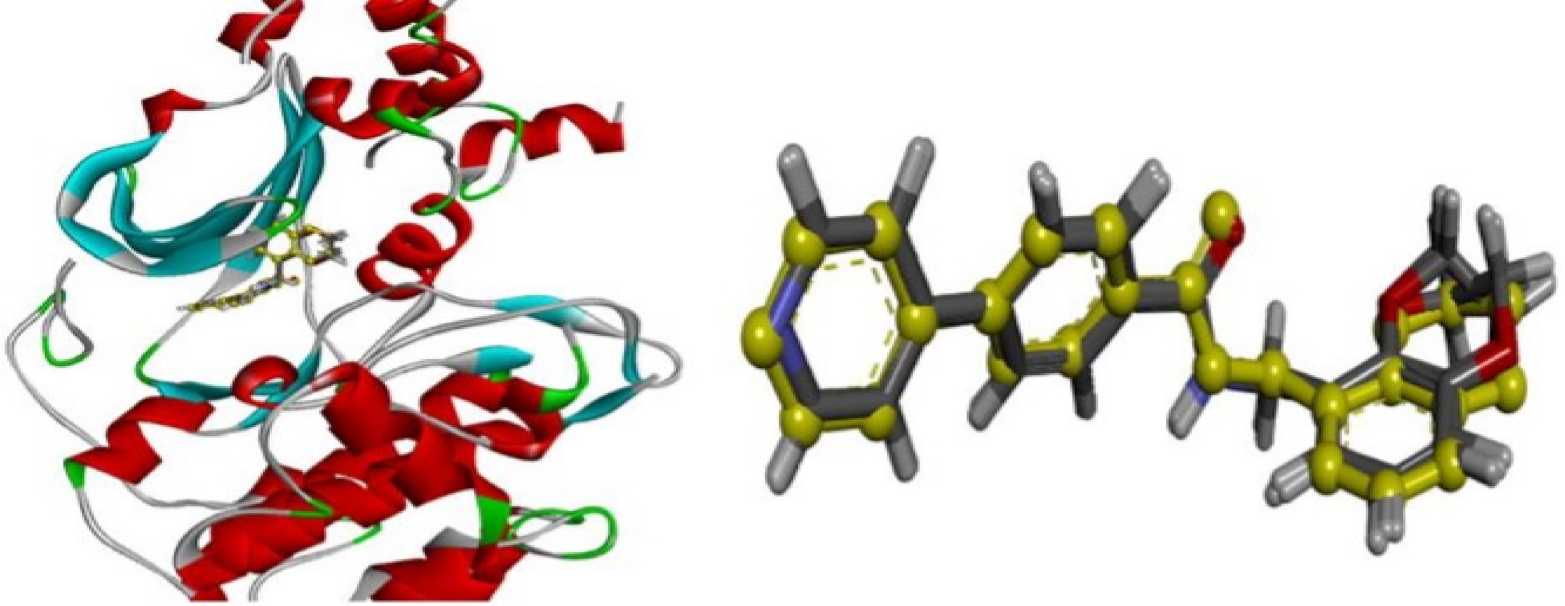
Molecular superposition diagram (yellow ball‐and‐stick model for eutectic ligands, grey for the lowest energy values of the four conformations).

After hydrogenating the eutectic ligand and minimizing the energy, CDOCKER redocks with the ROCK2 protein (6ED6) again. The top 10 positions with the lowest energy reserved were sorted in ascending order (CDOCKER_INTERACTION_ENERGY). The eutectic ligand in 6ED6 was copied into the docking results, and the RMSD values of the non‐hydrogen atoms in 10 positions were calculated with it as a reference, as shown in Table 1. The RMSD values of the top four lowest energy positions were all less than 0.5. The eutectic ligands were superposed in the four lowest energy positions, as shown in Figure 1. Eutectic ligands almost completely overlapped with the four positions, which means the CDOCKER method works well. The eutectic ligand structure was copied into the database, and 1969 matching molecules without bases were selected from the clinical approval trial database. After hydrogenation, the CHARMm Force field is used for energy minimization and Momanye Rone electrostatic charge is loaded on the molecules.

**TABLE 1:** The RMSD values of redocked conformations compared with coligand. The redocked conformations are arranged in ascending order of the CDOCKER_INTERACTION_ENERGY values.

Name	Reference	RMSD (Å)	CDOCKER_INTERACTION _ENERGY
Redock 1	Coligand 12	0.3183	−55.27
Redock 2	Coligand 12	0.2871	−55.08
Redock 3	Coligand 12	0.2710	−54.84
Redock 4	Coligand 12	0.3741	−54.80
Redock 5	Coligand 12	2.0849	−54.67
Redock 6	Coligand 12	0.8289	−49.23
Redock 7	Coligand 12	1.0113	−48.65
Redock 8	Coligand 12	3.7084	−48.21
Redock 9	Coligand 12	1.0102	−48.18
Redock 10	Coligand 12	3.7077	−48.17
Coligand 12	Coligand 12	0.0000	–

### 2.11 Statistical analysis

We used R software and GraphPad Prism 8 to perform statistical analyses, presented data as mean ± standard error (SD) and calculated statistical significance by the *t*‐test. *p* < 0.05 were considered statistically significant.

## 3 RESULTS

### 3.1 Screening of DEGs

**FIGURE 2:**
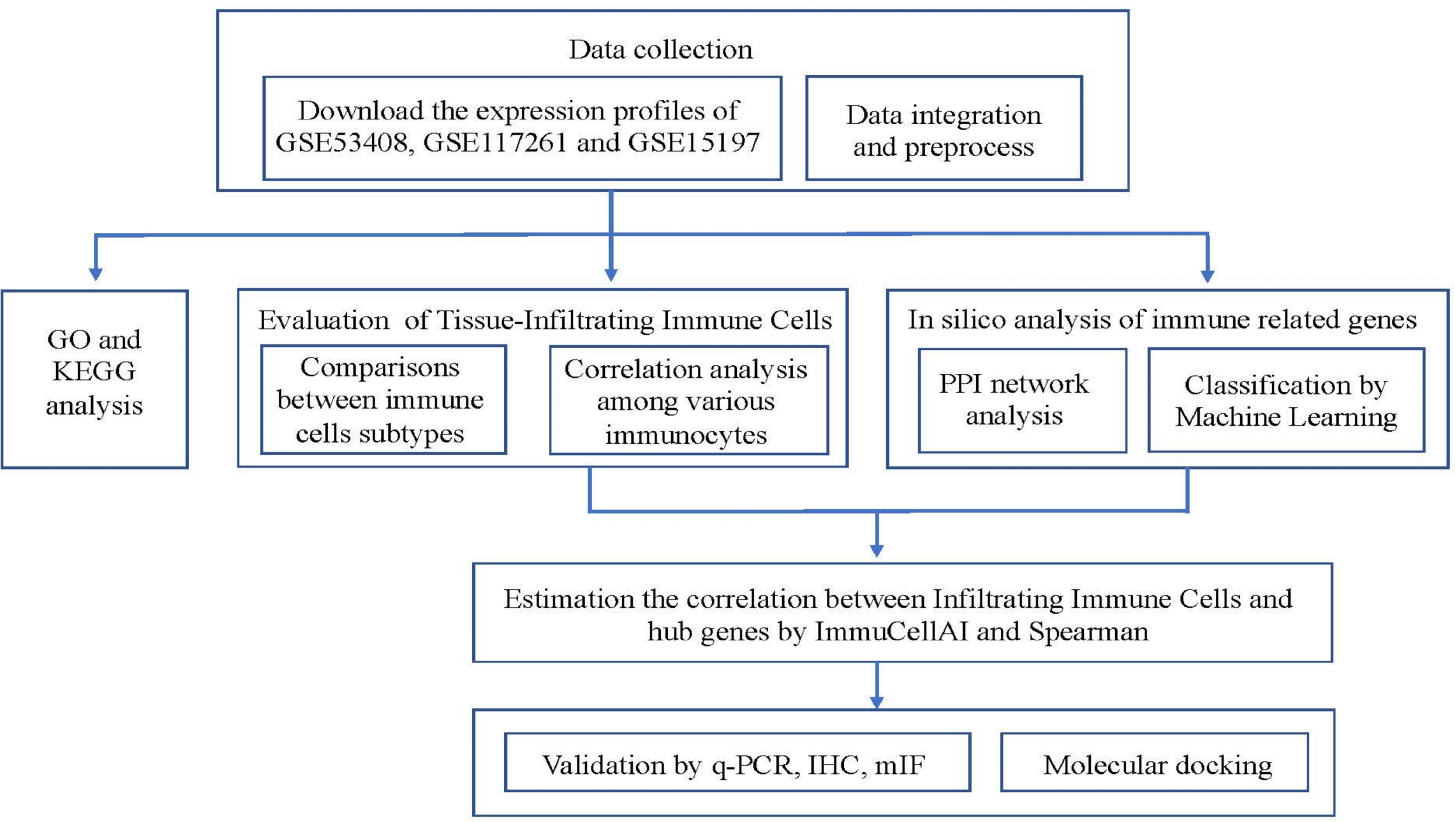
Study workflow.

**FIGURE 3:**
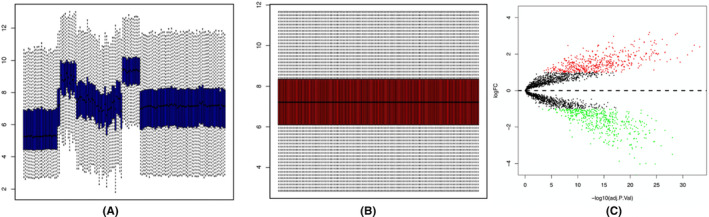
Screening of differentially expressed genes (DEGs) in pulmonary arterial hypertension (PAH) tissues compared with control tissues. (A, B) GSE53408, GSE117261 and GSE15197 datasets (49 normal controls and 84 PAH samples) were merged and normalized by sva package. (C) Volcano plots of all quantified genes in the gene expression profiles analysis of PAH and controls. Volcano map screened out statistically significant DEGs (*p* < 0.05 and |log_2_FC| > 1). Among these DEGs, there were 221 upregulated genes (represented by red dots) and 254 downregulated genes (represented by green dots).

A flowchart of the analysis procedures, including transcriptomic analysis and experimental validation, for this study was shown in Figure 2. There were 84 PAH samples and 49 normal controls from the GSE53408, GSE117261 and GSE15197 datasets merged and processed using the sva package in R, and 2758 genes were analysed and normalized in total (Figure 3A,B). Differential expression analysis was then performed using the limma Bioconductor package, with a threshold of |log_2_ fold change (FC)| ≥ 1 and *p* ≤ 0.05. This analysis identified 221 upregulated genes and 254 downregulated genes in PAH tissues when compared to normal samples, with a total of 475 DEGs identified, as shown in Figure 3C.

### 3.2 GO and KEGG pathway enrichment analysis

**FIGURE 4:**
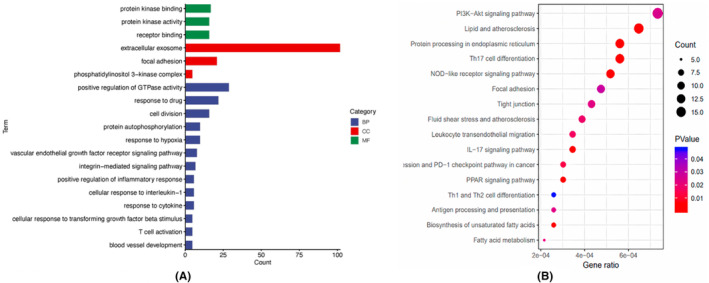
Functional enrichment analysis. (A) Enriched Gene Ontology (GO) terms for molecular function (MF), biological process (BP) and cellular component (CC). (B) Kyoto Encyclopedia of Genes and Genomes (KEGG) analyses of the pathways for differentially expressed genes (DEGs). The x axis shows the gene numbers and the colour of bar represents the *p*‐value. There were 16 pathways meeting the cutoff value of *p* < 0.05.

To identify the biological roles and signalling pathways related to the 475 DEGs, GO and KEGG enrichment analyses were conducted by the Cluster profiler R package. According to the GO enrichment analysis, most DEGs were significantly enriched in protein kinase binding, extracellular exosomes, positive regulation of GTPase activity, etc. (Figure 4A). KEGG pathway analysis revealed that most DEGs were primarily enriched in the PI3K‐Akt signalling pathway, lipid and atherosclerosis, protein processing in the endoplasmic reticulum, Th17 cell differentiation and the NOD‐like receptor signalling pathway (Figure 4B).

### 3.3 Estimating the characteristics of immune cells

**FIGURE 5:**
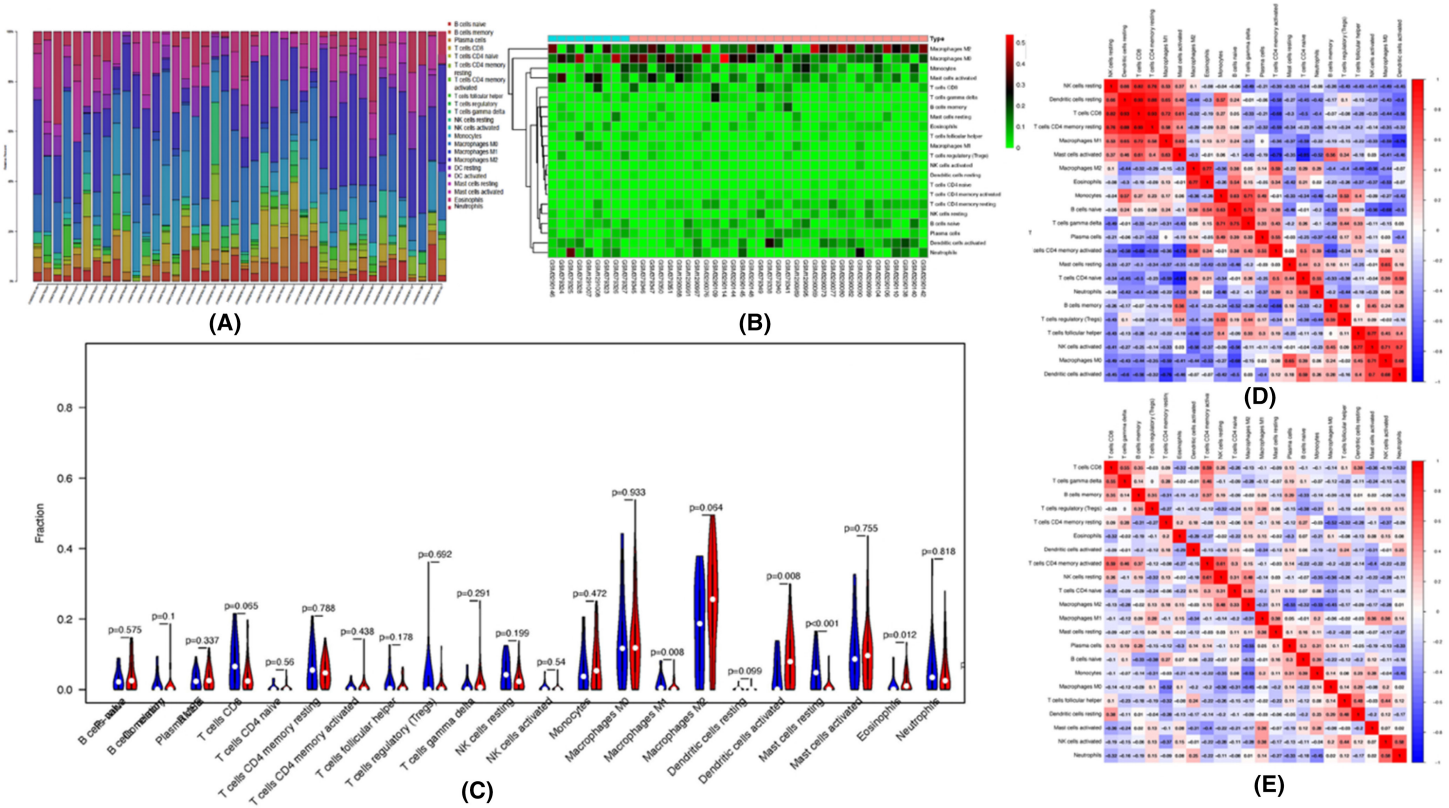
Analysis of immune landscape in the three datasets. (A) Histogram showing the abundance of immune cell subtypes in each sample (B) Heatmap of differentially cell types and individual samples. The expression profiles greater than the mean are coloured in red, and those below the mean are coloured in green. Blue, normal lung tissues; pink, PAH specimens. (C) violin plot showing the comparisons between immune cells in normal controls and PAH patients in the three datasets. Correlation matrix of the immunocyte proportions in Normal samples (D) and PAH samples (E). The colour of red and blue represent positive and negative correlations, respectively. The value represents the correlation coefficient.

Based on CIBERSORT analysis, the abundance of immune cell types in each sample was selected, and significant results (*p* < 0.05) from the three GEPs were presented in Figure 5A. The differences between the 22 types of immune cells and individual samples were shown in the heatmap with hierarchical clustering (Figure 5B). The analysis presented that naïve CD4 T cells were downregulated, whereas resting memory CD4 T cells and activated memory CD4 T cells were upregulated in the lung tissues of PAH patients. (Figure 5C).

The correlation analysis of PAH and controls further revealed the clues to the correlation among 22 immunocytes (Figure 5D,E). The results indicated that the correlation of immune cell subsets, including NK cells, resting dendritic cells, CD8 T cells and resting memory CD4 T cells, was strong in the normal group, while the correlation with PAH was weak. However, the correlation of CD4 T cells, including resting memory CD4 T cells, activated memory CD4 T cells and naïve CD4 T cells, remained significant compared with other cell subsets. Notably, the correlation of activated memory CD4 T cells with CD8 T cells and memory B cells was amplified in PAH.

### 3.4 In silico analysis of immune‐related genes

**FIGURE 6:**
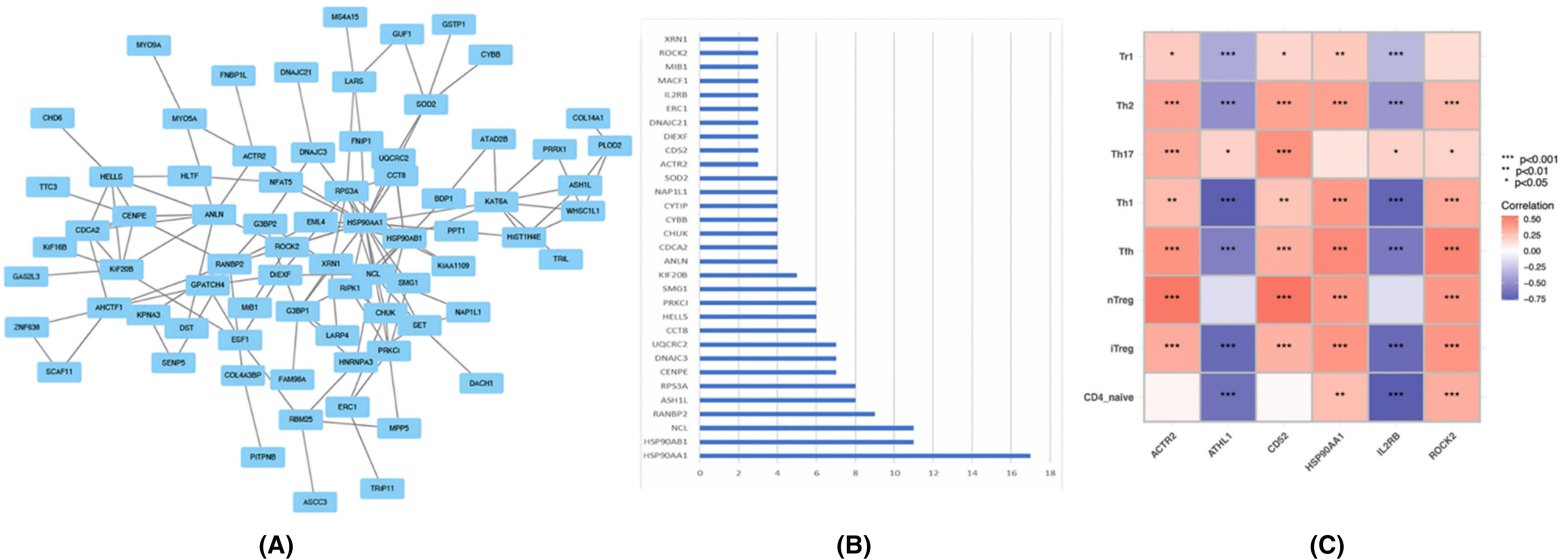
Immune‐related genes interaction and selection. (A) Immune‐related genes interaction network built by using STRING database and Cytoscape software. (B) The degree of the immune‐related genes with count of node more than three. (C) the correlation between various subtypes of immune cells and obtained genes by ImmuCellAI and Spearman.

The PPI network of processed DEGs determined with significant results (*p* < 0.05) was constructed using the STRING database and Cytoscape software. After removing the unconnected nodes, a PPI network that contained 58 genes was constructed (Figure 6A,B). Thirty‐one genes were considered hub genes, with an criteria of edge count beyond three. These genes were *HSP90AA1*, *HSP90AB1*, *NCL*, *RANBP2*, *ASH1L*, *RPS3A*, *CEVPE*, *DNAJC3*, *UQCRC2*, *CCT8*, *HELLS*, *PRKC1*, *SMG1*, *KIF20B*, *ANLN*, *CDCA2*, *CHUK*, *CYBB*, *CYTIP*, *NAP1L1*, *SOD2*, *ACTR2*, *CD52*, *DIEXF*, *DNAJC21*, *ERC1*, *IL2RB*, *MACF1*, *MIB1*, *ROCK2* and *XRN1*, which may play an important role in PAH progression.

To further explore the immune‐related gene signatures modulated during PAH development, PAH classifiers were built using machine learning algorithms. Before developing the models, Pearson correlation analysis was applied to filter the 31 hub genes. There were 20 genes with *p* < 0.05, which were used as the input variables for stepwise regression. Stepwise regression basically performs multiple regressions, removing the weakest relevant variables each time. After stepwise regression, six variables were left, which can best illustrate the distribution, including IL‐2rb, ROCK2, ATHL1, CD52, HSP90AA1 and ACTR2. The six genes were used as immune‐related genes to build NB, LR and SVM classification models. Table 2 shows the performance of the four models as estimated using fivefold random sampling and test set validation. The NB and LR classification models showed better performance, and both the precise and AUC of the three evaluation methods were beyond 0.918. ROC analysis also showed that the NB and LR algorithms with the six‐gene prognostic signature had high sensitivity and specificity for PAH classification.

**TABLE 2:** Performance of logistic regression, support vector machine (SVM) and naive Bayes models estimated by 10‐fold, random sampling and test validation.

Machine learning models	10‐fold cross‐validation		Random sampling		Test validation			
	AUC	Precision	AUC	Precision	AUC	Precision	Specificity	Sensitivity
Logistic Regression	0.938	0.904	1.000	0.865	0.950	0.906	0.851	0.829
SVM	0.948	0.951	0.692	0.832	0.917	0.921	0.873	0.771
Naïve Bayes	0.918	0.863	1.000	0.925	0.935	0.850	0.770	0.914

### 3.5 Estimation correlation between infiltrating immune cells and hub genes

According to CIBERSORT analysis, the differential cell type of the infiltrating immune cell population in PAH and controls was CD4 T cells. To estimate the correlation between various subtypes of CD4 T cells and the obtained genes, we analysed the normalized gene matrix by ImmuCellAI and Spearman. ROCK2, CD52, HSP90AA1 and ACTR2 were positively correlated with iTreg, nTreg, Tfh, Th1, Th17 and Th2, while ATHL1 and IL‐2rb were negatively correlated with naive CD4 T cell, iTreg, Tfh, Th1, Th17, Th2 and Tr1 (Figure 6C).

### 3.6 Validation of the density and distribution of infiltrating immune cells and immune‐related genes in MCT‐induced PAH through q‐PCR

**FIGURE 7:**
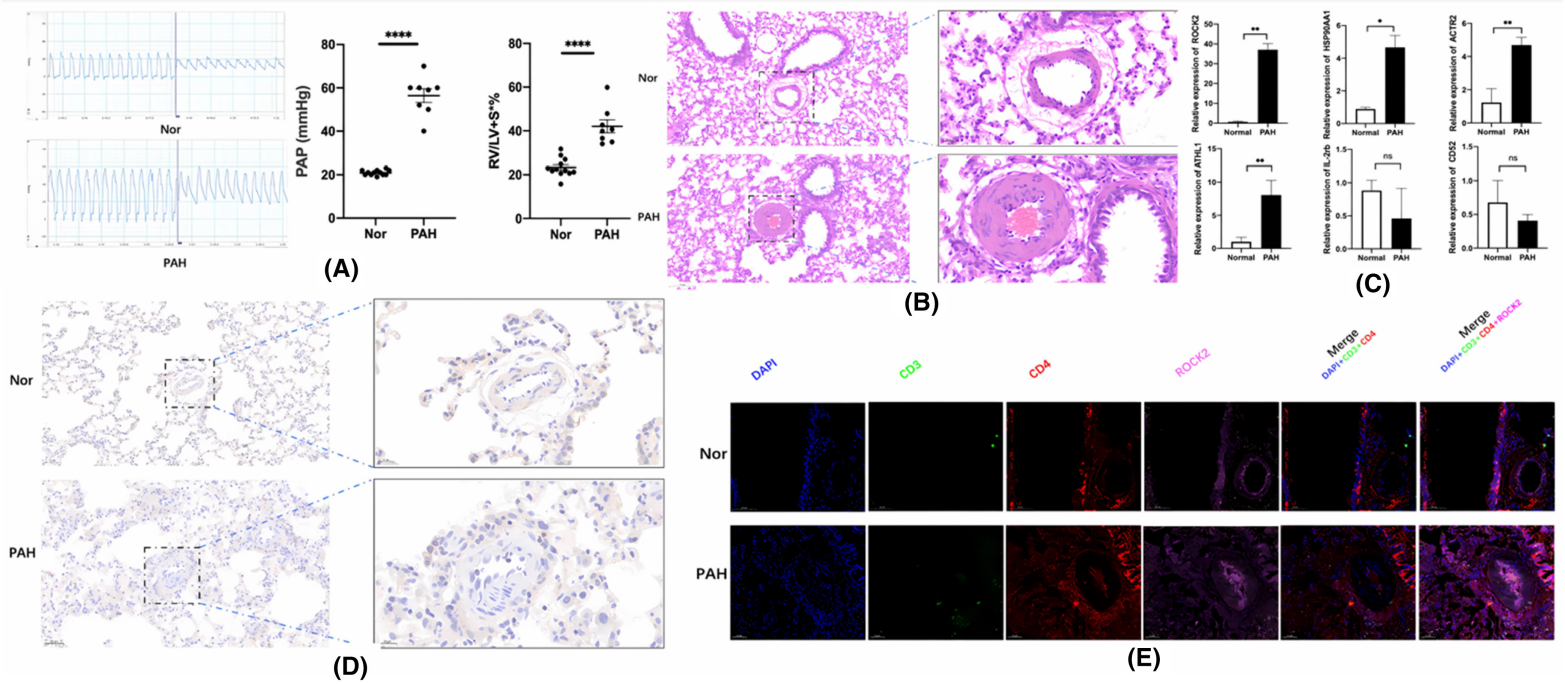
Validation by MCT‐induced PAH rat model. (A) Representative tracing of PAP in rat after MCT or Nor treatment and the mean values of PAP and Fulton index (RV/LV + S) in the two groups. ****p < 0.0001 versus Nor group. (B) Representative images of haematoxylin and eosin staining of lung sections from PAH rat or Nor rat. (C) The expression of ROCK2, ATHL1, HSP90AA1, ACTR2, IL‐2rb and CD52. *n* = 12 in Nor group, *n* = 8 in PAH group (MCT‐induced mortality in the model was about 33%). *p < 0.05, **p < 0.01, ns (no significance) versus Nor group. (D) IHC staining for ROCK2 from normal and PAH rats. (E) the density and distribution of infiltrating CD4 T cells (CD3+ CD4+)by multicolor immunofluorescence staining and detected the expression of representative hub gene (*ROCK2*) in successfully established PAH models by immunohistochemistry.

MCT‐induced PAH in rats is a well‐studied and published method to make preclinical models of PAH. However, there are currently no perfect animal models that replicate entirely human PAH. To investigate whether the pathological changes of PAH in human also occur in MCT‐induced rats and to validate the expressions of hub genes found in our analysis, we established a PAH model. After a 28‐day exposure to MCT, rats had a significant elevation in PAP (Pulmonary Artery Pressure) and RV/LV + S compared with those in control rats (Figure 7A). Meanwhile, MCT treatment resulted in pulmonary vascular remodelling, as evidenced by the increased media (Figure 7B). As shown in Figure 7C, the expressions of ROCK2, ATHL1, HSP90AA1 and ACTR2 were significantly upregulated, which was consistent with the results in the datasets of PAH patients. Meanwhile, we found that the expression of CD52 and IL‐2rb showed no difference between PAH models and controls. Then, we validated the density and distribution of infiltrating CD4 T cells by multicolor immunofluorescence staining and detected the expression of a representative hub gene (*ROCK2*) in successfully established PAH models by immunohistochemistry (Figure 7D,E).

### 3.7 Validation of the immune‐related genes in hypoxia‐induced PAH mice model

**FIGURE 8:**
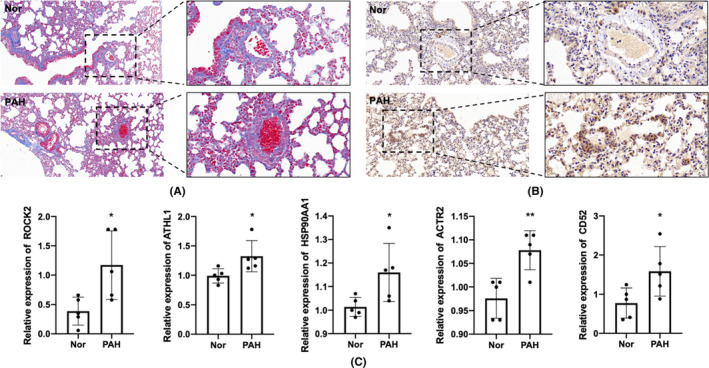
Validation through hypoxia‐induced PAH mice model. (A) Representative images of IHC of ROCK2 from PAH mice or Nor mice. (B) Representative images of Masson's trichrome staining of lung sections from PAH mice or Nor mice. (C) The expression of ROCK2, ATHL1, HSP90AA1, ACTR2 and CD52. *n* = 5 in each group. *p < 0.05, **p < 0.01 versus Nor group.

Following a 14‐day exposure to hypoxia, increased medial thickness, collagen deposition and muscularization of the distal arterioles were observed in hypoxia‐induced PAH mice model (Figure 8A), which is consistent with the model characteristics. And the increased expression of ROCK2 in the thickened and muscled microarteries of PAH mice was observed using IHC (Figure 7D,E). As shown in Figure 8C, the expressions of ROCK2, ATHL1, HSP90AA1 and ACTR2 were significantly upregulated, which was consistent with the results in the datasets of PAH patients and MCT‐induced PAH models. Additionally, the expression of CD52 was also increased, while IL‐2rb was not detected. These differences may be related to species differences and sample sizes.

### 3.8 Molecular docking

**FIGURE 9:**
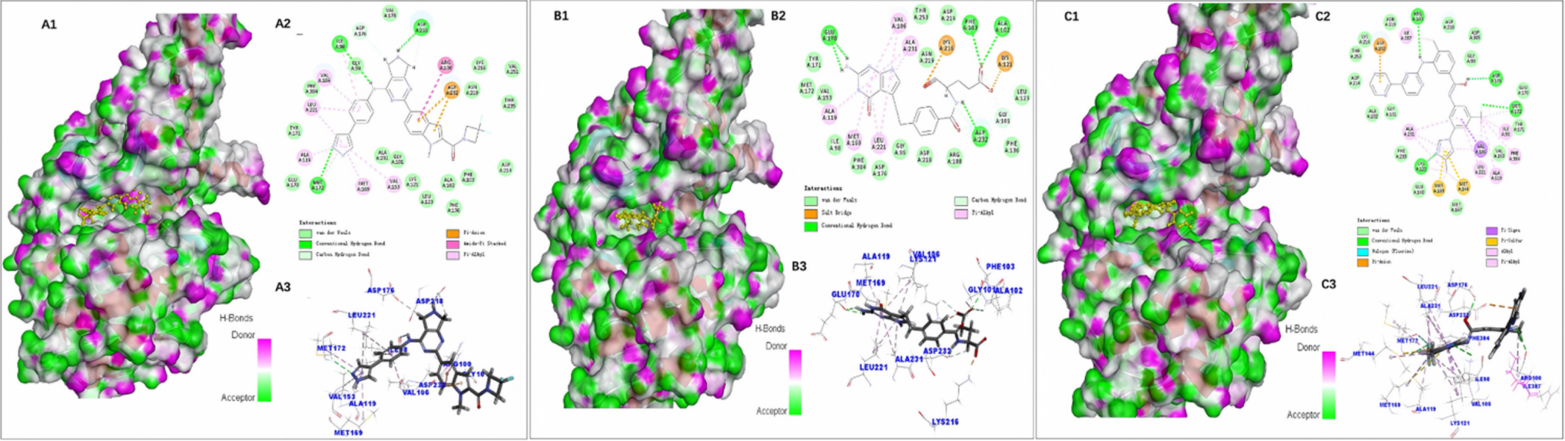
The results of the molecular docking between TDI01953 and ROCK2, pemetrexed acid and ROCK2, radotinib and ROCK2. The pyrazole ring in the drug skeleton structure forms a stable hydrogen bond with the hinge area MET172, the amino hydrogen of the indoline ring forms a hydrogen bond with ASP218, and which also forms a van der Waals interaction with ASP176. The hydrophobic ring of methyl indole and form hydrophobic interactions with ALA231, GLY101, LEU123, ALA102, PHE103, etc. (A1) The surfaces (H‐Bonds) of ROCK2 (6ED6) binding with compound TDI01953. (A2) Binding maps of TDI01953 with the active site of ROCK2. (A3) TDI01953 (Stick) interactions with amino residues (Line) of the active site of ROCK2. The amine group on the pyrimidine ring forms two hydrogen bonds with the hinge region MET172, which can also form hydrogen bonds with ALA102, PHE103 and ASP232. Two carboxyl groups can form a strong electrostatic interaction with the loop ring and two lysines (LYS121 and LYS216) in the catalytic region. (B1) The surfaces (H‐Bonds) of ROCK2 (6ED6) binding with pemetrexed acid. (B2) Binding maps of pemetrexed acid with the active site of ROCK2. (B3) pemetrexed acid (Stick) interactions with amino residues (Line) of the active site of ROCK2. The trifluoromethyl group of radotinib forms a hydrogen bond with MET172 of ROCK2, and which can also form a hydrogen bond with ASP176, ARG100 and LYS121; the pyrazine ring can form a π‐π stack with ASP232. (C1) The surfaces (H‐Bonds) of ROCK2 (6ED6) binding with radotinib. (C2) Binding maps of radotinib with the active site of ROCK2. (C3) Radotinib (Stick) interactions with amino residues (Line) of the active site of ROCK2.

Among the hub genes, *HSP90AA1* and *ROCK2* were most significantly upregulated in PAH tissues. It was reported that ROCK2 kinase inhibitors can reduce the phosphorylation of STAT3 and enhance the phosphorylation of STAT5, thereby downregulating overactivated T cells and rebuilding immune balance.^25^ In addition, ROCK2 inhibitors can also prevent tissue fibrosis and induce the regression of established fibrosis, which would relieve vasoconstriction. Here, we used ROCK2 as a potential therapeutic target and tried to screen targeted small‐molecule compounds through molecular docking. We used the binding site sphere (*x* = 26.896090, *y* = 47.022057, *z* = 52.444471, *r* = 9.798772) of 6ED6 for molecular docking by LibDock. The docked pose with the highest LibDock score was obtained for each compound. Each molecule retained 10 conformations with the lowest energy. A total of 11,860 conformations were retained from the docking results. Then we analyse the hydrogen bond, hydrophobic, electrostatic and other interactions between molecules and amino acid residues by using the analysed ligand pose in the receptor–ligand interaction module. Based on the result of analysed ligand poses, 638 conformations were retained by removing all conformations that formed hinge regions. Under the condition that each molecule retaining the lowest energy conformation and CDOCKER_INTERACTION_ENERGY value less than −40 kJ/mol, 79 molecules were screened by using the filter tool. Then we analysed the binding posture of each molecule to the docking pocket, focusing on the interaction with the aspartic acid (ASP176, ASP218, etc.) on the side of the pocket and whether the hydrophobic group can form a hydrophobic interaction with the hydrophobic region. Finally, 20 molecules were obtained by removing those poorly bounded molecules (Table 3). Figure 9. shows three randomly selected molecules docking results according to the classification of molecular structure.

**TABLE 3:** Docking analysis of virtual screening hits (key hydrogen bond interactions and CDOCKER _INTERACTION_ENERGY).

Compound ID	Hinge hydrogen bond		Other hydrogen bond		CDOCKER_INTERACTION_ENERGY
	No	Residue	No.	Residue	
Entrectinib	1	MET172	1	ASP232	−62.89
Dasatinib	1	MET172	1	LYS121	−54.82
TSU‐68	1	MET172	1	LYS121	−43.38
Lapatinib	1	MET172	1	ASP218	−64.05
Canertinib	1	MET172	4	LYS121 ASP218 ARG100 THR253	−59.41
Dacomitinib	1	MET172	–	–	−60.54
Neratinib	1	MET172	–	–	−53.97
Mubritinib	1	MET172	3	LYS121 GLY252	−53.67
Apatinib	1	MET172	–	–	−49.06
Radotinib	1	MET172	3	LYS121 ARG100 ASP176	−63.33
Pexidartinib	1	MET172	2	LYS121 ASP218	−47.14
Cerdulatinib	1	MET172	2	PHE101 LYS121	−57.55
Pemetrexed acid	2	GLU170	3	ALA102 PHE103 ASP232	−62.66
TDI01953	1	MET172	2	ILE98 ASP218	−56.89
TDI01910	1	MET172	2	GLY252 THR253	−47.84
TDI01999	1	MET172	3	ARG100 LYS216 ASP385	−47.31
PCI‐24781	2	GLU170 MET172	1	LYS121	−58.47
GDC‐0941	1	MET172	1	LEU122	−55.83
LDK378	1	MET172	3	ALA102 PHE103 ASP232	−67.10
Coligand	1	MET172	2	PHE103 LYS121	−55.27

## 4 DISCUSSION AND CONCLUSIONS

In our study, a total of 475 DEGs (221 upregulated and 254 downregulated) were identified in the transcriptional profiles in PAH tissues, mainly involved in pathways related to inflammatory responses or response to hypoxia. Using CIBERSORT analysis, we found that CD4 T cells were the differential cell type of the infiltrating immune cell population in PAH. Our findings suggest that the PI3K‐Akt signalling pathway was enriched with most differentially expressed genes, which is important in the development of PAH associated with CD4 T cells, including anti‐inflammatory Tregs and proinflammatory Th17 cells. The differentiation of Th cells from naive CD4 T cells is initiated by the stimulation of T‐cell receptors (TCRs) and associated costimulatory molecules upon antigen presentation, which leads to the activation of PI3K and mTORC.^26^, ^27^ PI3K–Akt–mTORC1 signalling positively regulates Th17 differentiation via multiple mechanisms.^28^ Natural Tregs develop via TGF𝛽‐independent mechanisms, and require TCR stimulation and CD28 costimulation signals that are controlled by PI3K, AKT and mTOR.^29^, ^30^, ^31^ As a new therapeutic idea, immune microenvironment and immunotherapy have been widely studied in various diseases.^32^ It is of great significance to understand the progression of PAH disease from this perspective. Using the PPI network and machine learning, six hub immune‐related genes were figured out. And then we demonstrated that the accumulation of CD4 T cells was infiltrated in and around vascular lesions, and the expressions of ROCK2, ATHL1, HSP90AA1 and ACTR2 were significantly upregulated in both MCT‐induced PAH models and hypoxia‐induced PAH mice. Among those with these validated genes, *ROCK2*, *HSP90AA1* and *ACTR2* were positively correlated with various CD4 T cells including Tregs, Tfh, Th1, Th17 and Th2, while ATHL1 were negatively correlated with them. At present, we know little about ATHL1. Although we know that PGGHG was the product of *ATHL1* gene,^33^ there were few studies on the function of ATHL1, which would be a novel research target. HSP90AA1 promotes autophagy through PI3K/Akt/mTOR pathway and inhibits apoptosis through JNK/P38 pathway. Autophagic abnormalities are observed in PAECs, PASMCs and in the RV of PH from both animal models and patients.^34^, ^35^ Although there had no direct evidence that HSP90AA1 plays a role in the process of PAH, HSP90AA1 is known to interact with endothelial nitric oxide synthase (eNOS), which is responsible for nitric oxide, a potent vasodilator.^36^ By stabilizing and promoting the activity of eNOS, HSP90AA1 may help maintain proper vascular tone and prevent excessive vasoconstriction, a key feature of PAH. ACTR2, an F‐actin modulator that were found to be essential for the formation of the actin cap, a cytoskeletal structure that is intimately associated to NE31, prevents nuclear aberrations and fragility with ensuing cGAS activation, while cGAS was characterized as a primary cytosolic DNA sensor that triggers IFNs and other inflammatory cytokines such as TNF‐a and IL‐6.^37^, ^38^, ^39^, ^40^ Rho‐ROCK2 signalling has been reported to be involved in hypoxia exposure, endothelial dysfunction, vascular smooth muscle cell proliferation, reactive oxygen species (ROS) production and inflammatory cell movement.^41^, ^42^, ^43^ Moreover, ROCK2 signalling pathway regulates the Th17/Tregs balance and controls profibrotic pathways.^44^ Toru Shimizu et al. has reported that the severity of hypoxia‐induced PAH was reduced in ROCK2 (+/−) mice, while it was enhanced in mice with ROCK2‐overexpressing transgenic (ROCK2‐Tg). In cultured mouse aortic vascular smooth muscle cells (VSMCs), ROCK2 (+/−) mice exhibited decreased migration and proliferation activities, whereas increased in ROCK2‐Tg mice.^45^ Feng Qiao et al. has reported that hypoxia increased the activity and expression of ROCK2 in pulmonary arterial endothelial cells (PAECs), and the stimulating effects of hypoxia on the proliferation of PAECs were attenuated with the ROCK inhibitor Y27632 or transfection with ROCK2 siRNA.^46^

Therefore, we took ROCK2 as a potential therapeutic target to find a list of 20 small‐molecule compounds that might modulate its activity through molecular docking simulations, including TDI01953, pemetrexed acid and radotinib. Pemetrexed is a folate antagonist used in the treatment of non‐small cell lung cancer and malignant mesothelioma.^47^ Radotinib, a high‐affinity BCR‐ABL1 inhibitor mainly used for resistance to imatinib, was approved in Korea for second‐line CML treatment in 2012.^48^ Notably, TDI01953 is an analogue of TDI01, which is a specific selective ROCK2 inhibitor undergoing clinical trials for pulmonary fibrosis and nonalcoholic steatohepatitis currently. Even though there is no listed ROCK2 inhibitor for the treatment of PAH, it has been evaluated in several clinical trials currently for various indications including, autoimmune diseases and cardiovascular disorders. Some of the ROCK2 inhibitors that have been tested in clinical trials include KD025 and Fasudil. KD025, a ROCK2‐specific inhibitor that reduces the expression of IL‐17 and fibrogenesis by inhibiting the ROCK2 signalling pathway.^44^, ^49^ A phase IIa clinical trial for the treatment of chronic graft‐versus‐host disease (cGVHD) showed quality of life improvements, corticosteroid dose reductions and limited toxicity.^44^ Additionally, it is worth mentioning that it can inhibit the increase of right ventricular systolic blood pressure in MCT‐induced rats.^50^ The non‐selective ROCK inhibitor Fasudil, approved for the treatment of cerebral vasospasm, can reduce the expression levels of endothelin‐1 and endothelin receptor, inhibit the proliferation of endothelial cells in pulmonary arterioles, and promote the generation of nitric oxide in rats with pulmonary hypertension caused by left heart disease.^51^ ROCK2 inhibitors show promise as a novel therapeutic approach for PAH. However, further clinical trials are needed to evaluate their safety and efficacy in larger patient populations.

Taken together, our study identified ROCK2, ATHL1, HSP90AA1 and ACTR2 as immune‐associated genes and provided insights into the reverse of the pulmonary vascular remodelling from the perspective of immunity and inflammation, which may be an effective and promising therapeutic strategy for PAH. There are some limitations to this study. First, this study employed microarray analysis, and all findings were predicated on gene expression levels. However, it should be noted that disparities at the genetic level of biomarkers do not inevitably translate to differences in protein‐level function and mechanism. The detailed molecular mechanisms associated with these four genes should be further addressed elaborately in the future. Second, it is still difficult to discern whether inflammation is the cause or initiating factor of pulmonary vascular remodelling or the consequence of the pulmonary high‐pressure microenvironment. Third, studies showed that pathologic specimens from PAH patients reveal an accumulation of inflammatory cells in and around vascular lesions, including macrophages, T and B cells, dendritic cells and mast cells.^52^ However, in this study, we only obtained the result of the differences in T cells, which may be due to the bias of the limited samples included in our data set and the cellular heterogeneity in lung tissues. Furthermore, this study provides preliminary evidence of a correlation between inflammation and immune and PAH pathogenesis; experiments need to be designed to further elucidate their mechanisms of action; and further in vivo data and clinical trials are warranted to investigate the role of inflammation and immune in PAH progression.

## AUTHOR CONTRIBUTIONS

**Xu He:** Conceptualization (lead); methodology (lead); project administration (lead); writing – original draft (lead). **Jiansong Fang:** Methodology (equal). **Mingli Gong:** Visualization (equal). **Juqi Zhang:** Data curation (equal). **Ran Xie:** Validation (equal). **Dai Zhao:** Visualization (equal). **Yanlun Gu:** Data curation (equal). **Lingyue Ma:** Visualization (equal). **Xiaocong Pang:** Conceptualization (equal); project administration (equal); supervision (equal). **Yimin Cui:** Conceptualization (equal); supervision (lead); writing – review and editing (equal).

## FUNDING INFORMATION

This research was funded by the National Natural Science Foundation of PR China, grant number (No. 82274015); National High Level Hospital Clinical Research Funding, grant number (No. 2022CX11) and National Key R&D Program of China, grant number (No. 2020YFC200830).

## CONFLICT OF INTEREST STATEMENT

The authors declare no conflict of interest. The authors declare that the research was conducted in the absence of any commercial or financial relationships that could be construed as a potential conflict of interest.

## Supporting information


Data S1:

## Data Availability

The expression profile data supporting this study were collected from [The Cancer Genome Atlas (TCGA) data portal (version July, 2019)]. The available public datasets from GEO database (https://www.ncbi.nlm.nih.gov/gds) were analysed in this study. We used molecular docking for screening Clinical and FDA‐approved Drug Library (https://www.apexbio.cn/discoveryprobetm‐clinical‐fda‐approved‐drug‐library.html). The processed data can be obtained from the corresponding author upon request.
