# Supplementary material for: Identification of immune‐associated signatures and potential therapeutic targets for pulmonary arterial hypertension
Source: J Cell Mol Med. 2023 Sep 27;27(23):3864–77. doi: 10.1111/jcmm.17962 (PMC10718157; doi:10.1111/jcmm.17962)
Supplement: Supplementary file 1 — Data S1: [file JCMM-27-3864-s001.docx]

Table 1. The sequences of q-PCR primers for lung tissues of MCT-induced rat model.

| **LOCUS** | **Names of PCR primers** | **Sequences of PCR primers** |
| --- | --- | --- |
| NM_008084.2 | M-GAPDH-Forward Primer | CCTCGTCCCGTAGACAAAATG |
|  | M-GAPDH-Reverse Primer | TGAGGTCAATGAAGGGGTCGT |
| NM_009072.2 | M-Rock2- Forward Primer | CGAGGTTCTGAAATCACAAGGA |
|  | M-Rock2- Reverse Primer | AGGTTCCTACAAGTGAATCTGCG |
| NM_010480.5 | M-Hsp90-A- Forward Primer | TTTACTCTGCCTATTTGGTTGCTG |
|  | M-Hsp90-A- Reverse Primer | CACAAAGAGAGTAATGGGATAGC |
| NM_001362899.1 | M-Actr2- Forward Primer | ACATTGTGCTTTCTGGAGGTTCT |
|  | M-Actr2- Reverse Primer | AGTTACACCAAGTTTCTCCAGCA |
| NM_008368.4 | M-Il2rb- Forward Primer | TCGTTCCCAGAGTCCCAGTCA |
|  | M-Il2rb- Reverse Primer | ACCAGGCGAAGGTTGTCAAA |
| NM_013706.2 | M-Cd52- Forward Primer | CAAAAACAGCACCTCCACCAA |
|  | M-Cd52- Reverse Primer | TTGGGATGTCTCTCGCTACTGAT |
| NM_145387.5 | M-ATHL1- Forward Primer | TTGATGGCTATGAGCGTGGA |
|  | M- ATHL1- Reverse Primer | ATCCAGCCCACAGCAAACA |

Table 2. The thermal protocol of q-PCR for lung tissues of MCT-induced rat model.

| Stage1 | Stage2（40 cycels） | Stage3 |
| --- | --- | --- |
| 95℃，30s | 95℃，15s | 65℃→95℃ |
|  | 60℃，30s | Collect one fluorescence signal per 0.5℃ temperature increase. |

Table 3. The sequences of q-PCR primers for lung tissues of hypoxia-induced mice model.

| **LOCUS** | **Names of PCR primers** | **Sequences of PCR primers** |
| --- | --- | --- |
| NM_008084.2 | M-GAPDH-Forward Primer | CCTCGTCCCGTAGACAAAATG |
|  | M-GAPDH-Reverse Primer | TGAGGTCAATGAAGGGGTCGT |
| NM_009072.2 | M-Rock2- Forward Primer | GGTTACTATGGGCGAGAATGTG |
|  | M-Rock2- Reverse Primer | CAAGTCGTACCTCCCTGTCTGT |
| NM_010480.5 | M-Hsp90-A- Forward Primer | TGAGGAAACCCAGACCCAAGA |
|  | M-Hsp90-A- Reverse Primer | GCTGGGAATGAGATTGATGTGC |
| NM_001362899.1 | M-Actr2- Forward Primer | ATCCAGGCAGTTCTGACTTTGTA |
|  | M-Actr2- Reverse Primer | CAGCAGAATGGTTGAAGGCA |
| NM_008368.4 | M-Il2rb- Forward Primer | AGGTCCAGGTGAGGGTCAAAG |
|  | M-Il2rb- Reverse Primer | GAAGAAGCCAGAAAAACAACCAAG |
| NM_013706.2 | M-Cd52- Forward Primer | CCTGCAGACTGTCCTGAACTC |
|  | M-Cd52- Reverse Primer | GTAGTGGCTTGTCCCAAGGAT |
| NM_145387.5 | M-ATHL1- Forward Primer | TGAGCAGGAGTTTCGCCAAT |
|  | M- ATHL1- Reverse Primer | AAGTGTCACCGAGTCCTTGGAAA |

Table 4. The thermal protocol of q-PCR for lung tissues of hypoxia-induced mice model.

| Stage1 | Stage2（40 cycels） | Stage3 |
| --- | --- | --- |
| 95℃，10min | 95℃，15s | 65℃→95℃ |
|  | 60℃，30s | Collect one fluorescence signal per 0.5℃ temperature increase. |
